# Development of a Reinforcement Learning Algorithm to Optimize Corticosteroid Therapy in Critically Ill Patients with Sepsis

**DOI:** 10.3390/jcm12041513

**Published:** 2023-02-14

**Authors:** Razvan Bologheanu, Lorenz Kapral, Daniel Laxar, Mathias Maleczek, Christoph Dibiasi, Sebastian Zeiner, Asan Agibetov, Ari Ercole, Patrick Thoral, Paul Elbers, Clemens Heitzinger, Oliver Kimberger

**Affiliations:** 1 Department of Anaesthesia, Intensive Care Medicine and Pain Medicine, Medical University of Vienna, 1090 Vienna, Austria; 2 Ludwig Boltzmann Institute for Digital Health and Patient Safety, 1090 Vienna, Austria; 3 Centre for Artificial Intelligence in Medicine, University of Cambridge, Cambridge CB2 0QQ, UK; 4 Department of Intensive Care Medicine, Laboratory for Critical Care Computational Intelligence, Amsterdam UMC, Vrije Universiteit, 1081 HV Amsterdam, The Netherlands; 5 Institute of Analysis and Scientific Computing, Department of Mathematics and Geoinformation, Technical University of Vienna, 1040 Vienna, Austria

**Keywords:** sepsis, corticosteroids, outcomes, artificial intelligence, reinforcement learning

## Abstract

Background: The optimal indication, dose, and timing of corticosteroids in sepsis is controversial. Here, we used reinforcement learning to derive the optimal steroid policy in septic patients based on data on 3051 ICU admissions from the AmsterdamUMCdb intensive care database. Methods: We identified septic patients according to the 2016 consensus definition. An actor-critic RL algorithm using ICU mortality as a reward signal was developed to determine the optimal treatment policy from time-series data on 277 clinical parameters. We performed off-policy evaluation and testing in independent subsets to assess the algorithm’s performance. Results: Agreement between the RL agent’s policy and the actual documented treatment reached 59%. Our RL agent’s treatment policy was more restrictive compared to the actual clinician behavior: our algorithm suggested withholding corticosteroids in 62% of the patient states, versus 52% according to the physicians’ policy. The 95% lower bound of the expected reward was higher for the RL agent than clinicians’ historical decisions. ICU mortality after concordant action in the testing dataset was lower both when corticosteroids had been withheld and when corticosteroids had been prescribed by the virtual agent. The most relevant variables were vital parameters and laboratory values, such as blood pressure, heart rate, leucocyte count, and glycemia. Conclusions: Individualized use of corticosteroids in sepsis may result in a mortality benefit, but optimal treatment policy may be more restrictive than the routine clinical practice. Whilst external validation is needed, our study motivates a ‘precision-medicine’ approach to future prospective controlled trials and practice.

## 1. Introduction

Sepsis represents a significant cause of morbidity and is responsible for 11 million deaths globally each year [1]. Defined as “life-threatening organ dysfunction caused by a dysregulated host response to infection”, sepsis is an umbrella term for a heterogeneous syndrome with many distinct phenotypes and wide variation in outcomes [2,3]. As a result, clinical trials have provided conflicting evidence concerning the benefit of specific therapies beyond source control, antibiotics, and maintenance of tissue perfusion [4,5].

Corticosteroids have been extensively investigated as a therapeutic option for sepsis ever since Cook et al. first advocated their use seven decades ago, but uncertainty regarding their optimal use nevertheless persists [6]. More recently, the case for corticosteroids in sepsis was based on the evidence of adrenal insufficiency accompanying critical illness [7]. Since diagnostic criteria for adrenal insufficiency are missing, identifying patients that should receive corticosteroids is challenging [7]. In addition, several studies have found that corticosteroids can lead to a faster resolution of shock but provided equivocal results concerning survival [8,9,10].

Currently, guidelines for the management of sepsis suggest using corticosteroids in septic patients with ongoing vasopressor requirement [5]. However, the optimal treatment regimen, particularly timing, duration, and dose of corticosteroids, is not known, and the clinical significance of potential adverse effects of corticosteroid therapy is unclear [5]. Identifying patients who are likely to benefit from corticosteroids is essential and attempts at personalizing corticosteroid therapy using novel approaches, such as machine learning and transcriptomics, have been reported [11,12].

Since interventional studies in sepsis are challenging due to the extreme heterogeneity of its phenotypes, machine learning could represent a complementary evaluation method for specific treatments using observational data. In essence, the aim is to construct an algorithm that can exploit clinician variances in treatment policy over a large dataset in a way that it is possible to find the effects of the treatment on similar patients at a given time. Reinforcement learning, one of the three primary machine learning branches, can be applied to this type of problem [13,14]. Reinforcement learning algorithms can serve as the foundation for decision support tools in intensive care, where decision making is based on sequential, highly granular data [15,16]. In brief, such algorithms attempt to find an ‘optimal’ policy that maximizes some reward function (for example survival), given a particular treatment strategy with a comprehensive description of the state of the patient at that time [13]. In the present study, we describe the development of a reinforcement learning algorithm to find the optimal approach to corticosteroid therapy in septic patients based on high-resolution clinical data from an intensive care database.

## 2. Materials and Methods

### 2.1. Data Sources and Data Processing

All data were queried from the AmsterdamUMCdb database. Approval was obtained for 3rd party re-use of AmsterdamUMCdb data for research from its steering group, and the research was conducted according to the data use agreement. Such a study of deidentified data is not subject to the need for ethical review. The ethical approvals for the AmsterdamUMCdb have been previously described [17]. AmsterdamUMCdb contains high-resolution clinical data related to 23,106 ICU admissions of 20,109 patients from 2003 to 2016 [17]. Patients with sepsis were identified based on the Sepsis-3 criteria2 Accordingly, patients with new organ dysfunction as indicated by either a SOFA score ≥ 2 at admission or an increase of 2 points or more in the SOFA score during the ICU stay, in the context of suspected infection as described in Supplemental Table S1, were included in the sepsis cohort [2,18,19]. Patients aged <18 years at the time of the ICU admission and patients who stayed in the ICU less than 24 h were excluded. The onset of the septic episode was considered the day the change in the SOFA score occurred and patients remained in the sepsis cohort until discharge or death.

In total, 281 variables were extracted, of which 277 input variables were coded as a multidimensional time series with a time resolution of 24 h. Every ICU day was considered separately, and only current measurements available at that timepoint were included in each data point. Only numeric variables represented in more than 2% of the data points were included. The imbalance resulting from missing data and the variable sampling rate were addressed by preprocessing: missing laboratory values were imputed using forward fill, while missing medication doses were set to 0. Overall, 17.93% of all input values were imputed. Numeric data were normalized to values between −1 and +1; for frequently sampled parameters (e.g., heart rate), the mean, the maximum, the minimum, and standard deviation were calculated, and for others (e.g., continuously administered drugs), the sum, i.e., the 24 h cumulative dose, was used as input instead. Therefore, the final number of extracted parameters increased to 379. The complete list of input features is provided in Supplemental Table S2.

### 2.2. Algorithm Development

Reinforcement learning is based on modeling a virtual decision-making ‘agent’ interacting with its environment described by a set of continuous states; the interaction between the agent and the environment predetermined as the action space (in this case, the finite number of treatment choices). At each step, the agent chooses an action, and the environment changes its state, returning a reward. The reward signal is used to train the agent, which gradually learns an optimal policy that maximizes return [20].

**Figure 1:**
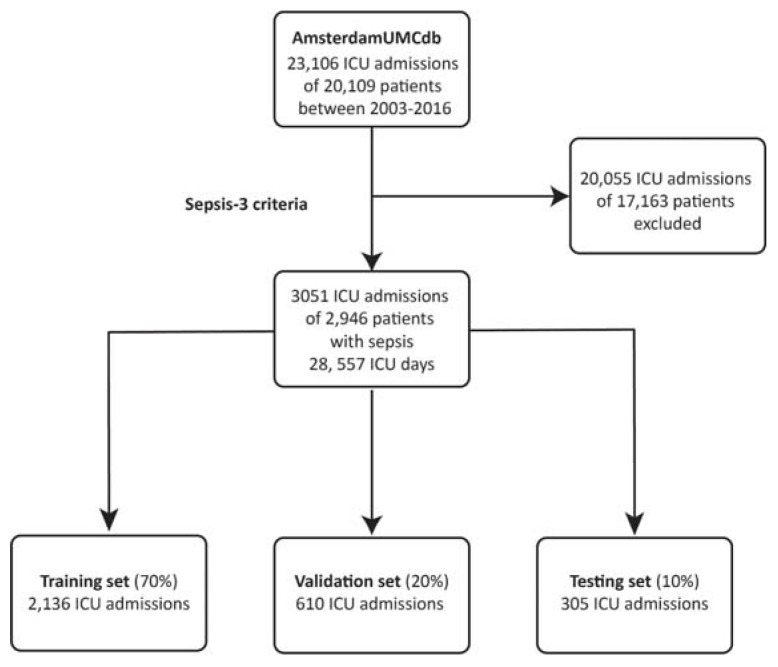
The Sepsis Cohort. Patients with sepsis from the AmsterdamUMC database were identified using the Sepsis-3 criteria. The sepsis cohort was randomly split in three distinct subsets used for training, evaluating, and testing the reinforcement learning algorithm.

We implemented a reinforcement learning algorithm, consisting of two distinct neural networks, based on the Markov Decision Process using the temporal difference actor-critic method able to suggest the optimal corticosteroid dose for each septic patients by retrospectively analyzing clinical data [20,21,22]. The dataset was randomly split into a training set, consisting of 70% of all patients, and two smaller datasets for validation (20%) and testing (10%) (Figure 1). The algorithm was trained on trajectories of successive patient states, where a state corresponded to a vector of all features within a 24 h period, other than mortality and the administered corticosteroid dose. The reward signal associated with each transition was related to the ICU mortality. The action space consisted of five discrete actions, defined by converting the cumulative 24 h dose of systemic corticosteroids to the equivalent dose of hydrocortisone and binning the resulting values: the null (‘no corticosteroids’) action and four dose ranges: 1–100 mg, 101–200 mg, 201–300 mg, and over 300 mg hydrocortisone [23]. A detailed description of the reinforcement learning model is provided in Supplemental File S1 and Supplemental Figure S1. The reinforcement learning algorithm was built using the TensorFlow 2.7 Python library [24].

### 2.3. Evaluation of the Algorithm

The reinforcement learning algorithm was initially evaluated by comparing the actual reward after concordant actions, i.e., when the actual treatment and the corticosteroid dose suggested by the agent were identical, with the reward after discordant actions in the testing set.

The performance of such reinforcement learning algorithms could not be directly evaluated by measuring the received reward of each action, since the reinforcement learning (evaluation) policy was different from the clinician (behavior) policy and the actual reward represented the performance of the clinician policy. We implemented a high-confidence off-policy evaluation (HCOPE) of the algorithm, a statistical method which compares the performance of the algorithm’s policy with a baseline, the performance of the clinician policy, and computes the probability that the algorithm’s policy has a performance below this baseline to select the best performing model. Using the clinician policy, a set of trajectories was generated and used to lower-bound the performance of the evaluation policy. The high-confidence off-policy evaluation (HCOPE) allowed for determining whether the 95% lower bound of the expected reward of the policy of the reinforcement learning agent exceeded the average reward of the clinician policy, i.e., the actual treatment the patients received [25,26].

Finally, we estimated the relative importance of each variable using a Layer-wise Relevance Propagation algorithm and ranked the input features of the RL algorithm according to their contribution to the agent’s decision [27]. To allow for comparison between the relevance of the input features of agent’s policy and the clinical practice, we developed a random forest model using the Scikit-learn Python library that predicts the clinicians’ policy, simulating the clinician behavior, and we ranked the clinical variables supporting the average clinician behavior according to the parameters of the fitted model [28].

## 3. Results

A total of 3051 ICU admissions at the Amsterdam UMC corresponding to 2946 distinct patients were included (Figure 1).

Repeated admissions to the ICU, both remote and during the same hospital stay, were included if they met the sepsis definition and were analyzed as independent ICU stays. 1395 admissions were associated with vasopressor use and lactate values >2 mmol/l during the ICU stay, therefore meeting the criteria for septic shock. The cumulative length of stay from the onset of sepsis until ICU discharge was 28,557 days corresponding to as many data points. The training dataset comprised 2136 randomly selected ICU admissions, leaving a total of 610 and 305 admissions in the evaluation and testing datasets, respectively (Figure 1). Patients’ characteristics are summarized in Table 1.

**Figure 2:**
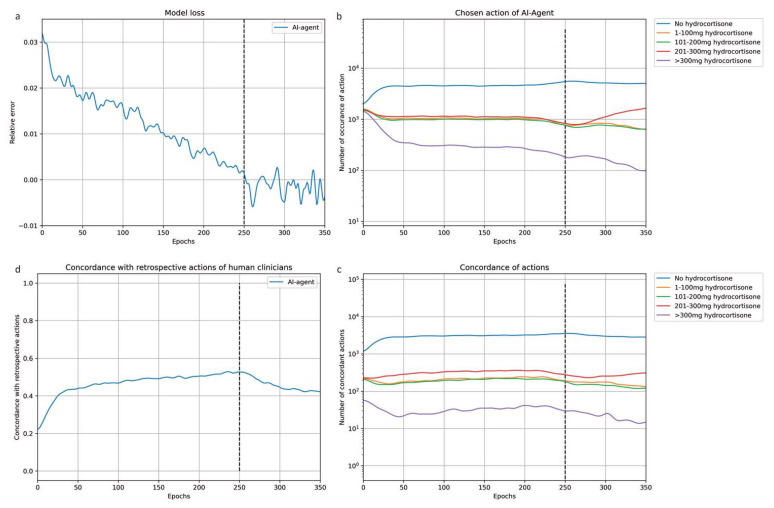
Training process of the virtual agent. Figure 2 shows how the performance and the behavior of the RL agent changed during the training process. On the X-axis, the number of epochs, i.e., how many times the algorithm had worked through the learning dataset, since the beginning of the training is displayed. The vertical dotted line marks the end of the training process. (**a**) The decrease in the relative error, which reflects the accuracy of the model’s output, during the training process. (**b**) The number of occurrences for each action suggested by the algorithm during training is displayed in the (**b**). All five possible actions are equally represented at the beginning of the training. After 50 epochs, the algorithm’s tendency to withheld corticosteroids becomes obvious. (**c**) The increasing overall agreement between the RL policy and the actual historic treatment. (**d**) The number of occurrences when agreement between the RL policy and the retrospective treatment was reached is displayed across the five possible actions in (**b**).

The relative error of the actor-critic model decreased over the training steps and converged after 250 epochs at 0.044 of the initial relative error (Figure 2a). The concordance between the virtual agent’s action and the retrospective action by ICU physicians started at 22%, which was the expected value considering the dimension of the action space (five possible actions). The overall agreement between the virtual agent and the human clinicians reached 63% after convergence (Figure 2c). Similarly, the probabilities of choosing each action from the action space were equal initially. Over the training epochs, the virtual agent increasingly tended towards withholding corticosteroids. After convergence, in 65% of ICU days, the agent chose to withhold corticosteroids, and in patients where corticosteroids were prescribed, the suggested dose was low (Figure 2b). In contrast, the human clinicians prescribed corticosteroids in 45% of data points. Although the virtual agent displayed a tendency towards passive behavior, in 49% of the cases where the agent chose to administer glucocorticoids, the ICU physicians acted concordantly.

**Figure 3:**
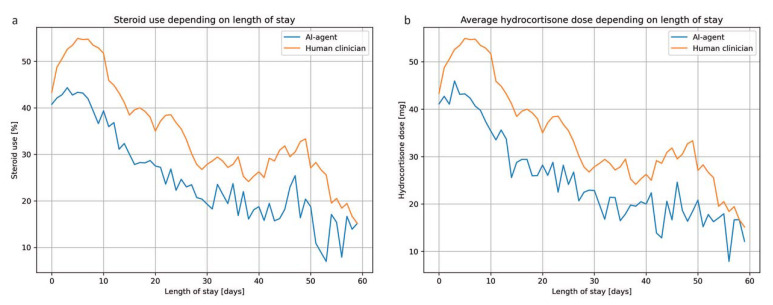
Comparison of corticosteroid use between ICU physicians and the RL agent. Use of corticosteroids as percentage of patients receiving corticosteroids (**a**) and average cortisone dose (**b**) is compared between the historic treatment in the ICU and the RL policy after adjusting for the ICU length of stay. Both ICU physicians and the RL agent tend to prescribe corticosteroids during the early phase of the septic episode. Notably, the RL policy is more restrictive compared to the actual treatment the patients received.

In the testing dataset, the treatment suggested by the virtual agent matched the retrospective action by ICU physicians in 59% of the data points. The agent’s tendency to prescribe less corticosteroids was also confirmed in the testing dataset: corticosteroids were withheld in 62% of the ICU days, compared to 52% according to the ICU physicians. Accordingly, the average daily corticosteroid dose prescribed by the virtual agent was lower (Figure 3). Both ICU physicians and the RL agent tended to prescribe corticosteroids in the early phase of the septic episode and corticosteroid use dropped sharply after 10 days (Figure 3).

**Figure 4:**
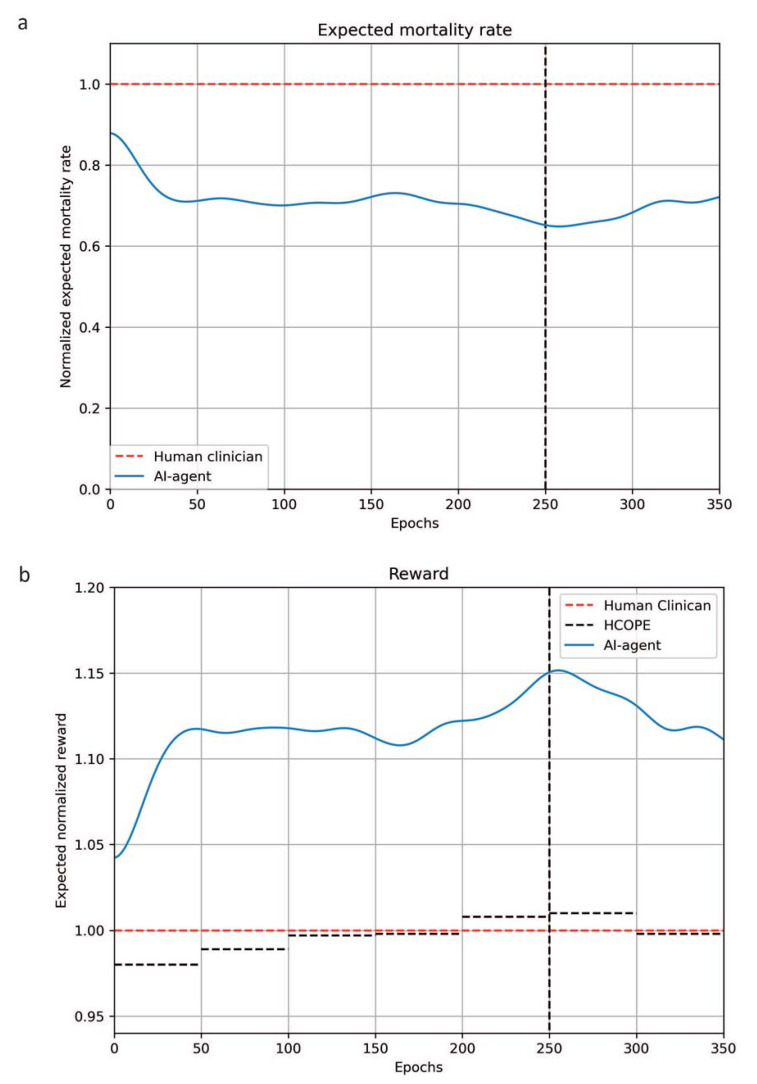
Comparison between the evaluation (RL) policy and the behavior policy (the actual treatment). (**a**) The change in the normalized expected mortality rate across training epochs, (i.e., the number of iterations or how many times the algorithm had worked through the learning dataset, since the beginning of the training) is represented in Figure 5a. (**b**) The 95% lower bound of the normalized expected reward of the RL policy (black dotted line) determined by high-confidence off-policy evaluation compared to the estimated reward of the clinician policy (red dotted line) is shown in (**b**).

The ratio between the reward of the agent’s policy and the clinicians’ policy increased over the training process and high-confidence off-policy evaluation (HCOPE) demonstrated that the 95% lower bound of the expected average reward for the agent’s policy was higher compared to the average reward for the historical decisions by clinicians after 200 epochs (Figure 4). Accordingly, the normalized expected mortality rate decreased and was lower than 0.7. Overall, when patients from the testing set received the same glucocorticoid therapy as suggested by the RL agent, mortality was lower: the mortality across all ICU days, i.e., the ICU days that eventually result in patient’s death, when the decisions made by the RL agent and the ICU physician were identical was 22.38% compared to 28.33% in case the actions were different. This finding was consistent both when the RL agent withheld corticosteroids (25.85% of the data points compared to 32.22%) and when the RL agent suggested using corticosteroids (33.02% of the data points compared to 34.27%).

**Figure 5:**
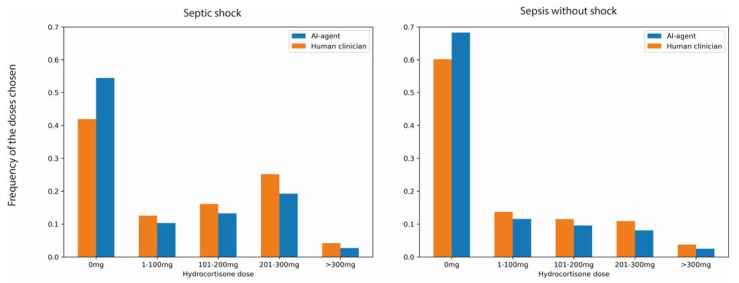
Comparison between the RL and physician policy in patient states grouped by septic shock criteria.

We modeled the retrospective treatment policy by the ICU physicians using a random forest model that predicted the clinicians’ treatment decisions. The micro-average multiclass Area under the Receiver Operator Characteristic Curve for the random forest model was 0.8 (Supplemental Figure S2). The most relevant input features underlying the decisions of the reinforcement learning algorithm and the random forest model, respectively, are presented in Supplemental Tables S3 and S4 and Supplemental Figure S3. Both algorithms relied on vital parameters and laboratory values to determine the optimal treatment policy. However, vasopressor use and PEEP were distinctly more relevant for the clinician policy. Accordingly, although the reinforcement learning agent was consistently more restrictive compared to human clinicians, the difference is more obvious in patients who met the criteria for septic shock (Figure 5).

## 4. Discussion

We present a reinforcement learning algorithm trained to optimize the corticosteroid treatment strategy for a specific patient state in critically ill patients with sepsis. The novelty of our approach is that it potentially enables an individualized therapy to improve a highly relevant outcome based on clinical parameters routinely collected in the ICU. The goal of our reinforcement learning algorithm, determined by the reward signal, was to minimize mortality. Indeed, in the testing dataset, ICU mortality was the lowest in patients who received a treatment identical to the action suggested by the algorithm. Off-policy evaluation confirmed that the algorithm performed well within the given environment and even outperformed the clinician policy in the validation dataset.

Currently, the rationale for corticosteroids in sepsis is based on several studies suggesting faster resolution of shock in septic patients who require vasopressors despite adequate fluid resuscitation [5]. While earlier studies showed a mortality benefit, this was not consistently confirmed in subsequent trials [8,9,29,30,31,32]. This led to frequent changes in the clinical practice to accommodate new, often conflicting evidence, which have been likened to a “swinging pendulum” situation [30]. The most recent guidelines for the treatment of sepsis suggest corticosteroids as early as 4 h after the initiation of treatment in patients who require vasopressors. In the testing subset of our sepsis cohort, where 45.7% of patients met the criteria for septic shock, corticosteroids were suggested by the virtual agent in 38% of the ICU days. Conversely, ICU physicians used corticosteroids in 48% of the data points, yet only in 49% of the cases where the reinforcement learning agent suggested using corticosteroids, the actual treatment prescribed in the ICU was concordant. This difference may be a result of at least two factors. First, the reward signal used for training was related to the mortality and the reinforcement learning agent aimed to maximize survival. Second, corticosteroids have been historically reserved for patients who require more vasopressors and have higher severity of disease and, therefore, worse outcomes. Indeed, the random forest model we developed to simulate decision making by the ICU physicians showed that blood pressure and vasopressor use were most consistently associated with corticosteroid use. Furthermore, due to the retrospective nature of our study, we expected that the association between higher severity scores and corticosteroid use in the database would translate in a bias of the RL policy towards the null action.

We identified patients from the database with sepsis algorithmically, and this required a pragmatic operationalization of the Sepsis-3 criteria, using a data-driven approach, instead of relying on coding data to be defined [2,19]. This method has been used before and has the advantage of being more reliable; more reproducible; and therefore, appropriate for epidemiological or database studies [33]. These operational criteria can provide consistent estimates of the sepsis incidence over longer periods, despite its inherent limitations, such as the assumptions about suspected infections being confirmed, pre-admission organ function, and the impact of the caregivers’ decisions on the SOFA score [33,34,35].

Although traditionally, artificial intelligence algorithms have been often compared to a black box, several methods are available to provide insight into which variables contributed most to the algorithmic decisions [36]. We ranked the input features based on relevance, showing that our model was explainable and valid from a clinical standpoint and that the agent relied on plausible clinical variables to make its decisions. If the random forest model accurately simulates the decision-making process by ICU physicians, comparing the relative relevance of the input features between the reinforcement learning algorithm and the random forest model can reveal how a treatment policy can be developed to maximize ICU survival contrasts with actual care. Unlike current clinical practice, where refractory shock is the single most important factor considered to prescribe corticosteroids, vasopressor requirements and lactate only had a limited influence on the reinforcement learning policy while being highly relevant for the clinicians’ policy. Similarly, the time elapsed since the onset of sepsis ranked distinctly higher amongst input features for the historical treatment by ICU physicians compared to the reinforcement learning treatment. These findings confirm the usual practice of prescribing corticosteroids early for patients in septic shock [5]. Interestingly, the machine learning policy resulted in a similar corticosteroid use pattern, characterized by an abrupt fall in steroid use after the 10th day since onset without explicitly relying as much on the time elapsed from the onset of sepsis. Conversely, total protein in cerebrospinal fluid (CSF) and the standard deviation of the heart rate ranked higher among the input parameters of the virtual agent only. It might seem surprising that a parameter that is rarely sampled is highly relevant for the output of the algorithm. Although corticosteroids are recommended for prevention of neurological sequelae in patients with bacterial meningitis, they have no effect on mortality [37]. Alternatively, lumbar puncture might be performed as a part of the work-up in patients with fever of unknown origin and subtle neurological symptoms [38]. In either case, since non-missing values are highly suggestive of a neurological diagnosis, informative missingness might explain its relevance for the reinforcement learning policy.

Arterial blood pressure, leucocyte count, serum sodium, and blood glucose levels were similarly influential in both algorithms. These findings seem biologically plausible, given the essential role of corticosteroids in regulating glucose metabolism and electrolyte homeostasis [39]. Corticosteroids also potentiate the effects of catecholamines and mobilize neutrophils, leading to leukocytosis and neutrophilia [40,41]. It is reasonable that clinical variables related to the physiological effects of corticosteroids could help guide therapy in septic patients by accurately predicting their effects in specific patient states. However, these results must be interpreted cautiously. The method we used to rank input variables estimates the overall contribution of all variables to the output of the model. Furthermore, unlike traditional statistical modeling, neural networks are less suitable for determining relationships between variables. Finally, all input variables were normalized between −1 and +1, and the relation between the normalized values, the actual values, and the reference range for each variable was determined by the variable’s distribution and is not obvious or readily interpretable for clinicians.

We acknowledge several limitations of our study. First, we used a single database to develop our algorithm and our findings have not been externally validated, which considerably limits the clinical applicability of the model. Like most of the artificial intelligence research in the intensive care, our study is in the prototype phase, and broad implementation remains a distant goal [42] Although machine learning models could be transferred across ICUs, moving these models to the bedside proves challenging [42]. Artificial intelligence holds great promise to enhance the practice of intensive care and the management of sepsis in the ICU; however, the current state of AI in intensive care does not support its routine use due to regulatory reasons, but also because uncertainty surrounds how these models could be included in daily practice, and good prospective studies still need to be included. Second, data used to train and test the model originate from a single medical center over several years. Changes in the best care practices over time and differences between local policies concerning ICU admission and sepsis management might result in relevant heterogeneity of the sepsis cohort and the outcomes. However, the aim of this study was to create an algorithm that can exploit these differences to derive an optimal treatment policy by analyzing several different suboptimal policies. Third, data were anonymized, and in the process, all notes were removed. Consequently, we could not account for the withdrawal of life-sustaining therapies. Fourth, by using a 24 h step to model the patients’ trajectories, our model artificially creates data points that encompass more data than are available to the clinician at any given time. We considered the time resolution of 24 h and the action space defined as the cumulative 24 h dose of corticosteroids due to several reasons, since this approach allowed us to compare different treatment regimens, using different substances, doses, and intervals. Furthermore, in our experience, therapy goals and some therapeutic measures for the next 24 h are defined during the ICU rounds, once daily. Therefore, modelling clinical data as time-series data with a resolution of 24 h resembles, to some extent, clinical practice.

Decision making in the ICU typically takes place during the once-daily rounds and the cumulative 24 h dose allows for different treatment regimens to be compared regardless of substance and timing. Finally, we analyzed all clinical data from onset of sepsis until discharge from the ICU, which most likely covers a significantly longer period than the duration of the septic shock. However, clearly delineating between the acute critical illness, and subsequent organ dysfunction and persistent critical illness does not seem feasible in the context of the present study.

## 5. Conclusions

We developed and evaluated a reinforcement learning algorithm that used clinical data to derive the optimal corticosteroid therapy aimed at improving mortality in patients with sepsis. The algorithm performed well in the testing dataset, and the reinforcement learning policy was associated with a lower mortality than the clinician’s policy. Due to the exploratory nature of our work, future research focusing on external validation of the model is required before prospective evaluation at the bedside. Our model suggests that a more targeted and individualized, reinforcement learning-driven approach to corticosteroids is possible and motivates prospective evaluation of treatment scenarios beyond refractory shock.

## Figures and Tables

**Table 1:** Summary of patients’ characteristics. Each ICU admission is considered separately.

Characteristics	Summary (Total)	Summary (Survivors)	Summary (Non-Survivors)
Total number of ICU admissions	3051	2336	715
Male sex, No. (%)	1758 (57.6%)	1353 (57.9%)	405 (56.6%)
Age group (years), No. (%)	--	--	--
18–39	342 (11.2%)	303 (12.9%)	39 (5.4%)
40–49	322 (10.5%)	265 (11.3%)	57 (7.9%)
50–59	518 (17.0%)	414 (17.7%)	104 (14.5%)
60–69	757 (24.8%)	591 (25.2%)	166 (23.2%)
70–79	709 (23.2%)	506 (21.6%)	203 (28.3%)
>80	403 (13.2%)	257 (11%)	146 (20.4%)
Highest SOFA score during the ICU stay, Median (IQR)	10 (6)	9 (6)	13 (6)
Sofa score at sepsis onset, Median (IQR)	9 (6)	8 (5)	11 (7)
Septic shock, No. (%)	1395 (45.7%)	845 (36.1%)	550 (76.9%)

## Data Availability

Access to the dataset used in this manuscript may be requested from Amsterdam Medical Data Science (https:/amsterdammedicaldatascience.nl/ accessed on 1 January 2021).

## Supplementary Materials

The following supporting information can be downloaded at https://www.mdpi.com/article/10.3390/jcm12041513/s1, Supplemental Table S1: Diagnosis of sepsis. Supplemental Table S2: Input features included in development of the algorithm. Supplemental Figure S1: Development of the RL Algorithm. Supplemental Figure S2: Micro-average ROC curve of the SVM algorithm. Supplemental Table S3: The most relevant predictors of the clinicians’ policy according to the SVM algorithm ordered from the lowest to highest rank. Supplemental File S1: The 20 most relevant input features for the RL and random forest models [43,44,45].
