# Supplementary material for: Development of a Reinforcement Learning Algorithm to Optimize Corticosteroid Therapy in Critically Ill Patients with Sepsis
Source: J Clin Med. 2023 Feb 14;12(4):1513. doi: 10.3390/jcm12041513 (PMC9961939; doi:10.3390/jcm12041513)
Supplement: Supplementary file 1 [file jcm-12-01513-s001.zip › Supplemental File S1.pdf]

1 Background

1.1 Definitions i n R einforcement Learning

Reinforcement learning (RL) is a machine learning method for solving near-optimal highly-dimensional control problems, which may not be analytically tractable. Unlike supervised learning, reinforcement learning does not require a data set of analyzed sample data. Instead, an agent interacts with an environment $\mathcal{E}$ and independently learns a strategy to maximize a reward r generated by this environment. The agent receives no information or instructions about the optimal strategy, but only the reward. Before specific algorithms can be discussed, certain basic terms must be explained. The following definitions of the basic terms of RL are based on the discussion in reference e [43].

> The policy (strategy) $\pi(a|s) = \mathbb{P}(A_t = a|S_t = s)$ defines the behavior of the agent at a certain point in time. This is achieved by mapping a distribution of actions $a \in \mathcal{A}(s)$ to every possible state $s \in \mathcal{S}$. The complexity of the policy depends strongly on the environment. In the simplest cases the policy can be represented by a table of all possible states. However, it is mostly stochastic in nature, which can demand enormous computing power for complex applications. The policy can be seen as the core of reinforcement learning, since the policy alone is sufficient to determine the agent's behavior.
>
> The reward signal defines the final target policy of the reinforcement learning problem. After each step, the environment sends a real number, the reward r , to the agent. The agent's only task is to maximize the reward over all episodes, which is described by the return: The return is the discounted, cumulative future reward from time t until the end of the episode, $G_t = \sum_{k=0}^{\infty} \gamma^k r_{t+k+1}$ where T is the terminal time step and $\gamma \in (0, 1]$ is a *discount* factor that determines the degree to which immediate rewards are favored over delayed rewards. The reward depends on the environment's current state and the agent's current action. The agent can only influence the reward signal by selecting certain actions. Therefore, the reward signal represents the primary basis for changing the policy. If a selected action results in a low reward, the policy is changed so that the agent acts differently for the same initial situation in the future.
>
> The value function $V_\pi(s) \doteq \mathbb{E}[G_t|S_t = s]$ for the policy π will indicate the most profitable in the long run. In principle, the value of a state is equal to the total reward an agent expects to accumulate starting from a state in the future. In other words, the reward is the intrinsic and immediate desirability of a state. On the other hand, the value of a state describes the desirability in the long run since it considers the rewards that will be achieved in subsequent states. Values are, therefore, predictions of rewards and are taken into account in the decision of the action more strongly than the reward itself. The agent seeks an action with maximum value, not with maximum reward. However, estimating the value is significantly more difficult than the reward. Rewards can be taken directly from the environment. Values must be estimated from observation sequences. The method of accurately estimating the value of a state is classified as the most important component of reinforcement learning.

Reinforcement learning algorithms are based on the Markov Decision Process (MDP), comprising five main elements: state space $\mathcal{S}$, action space $\mathcal{A}$, environment $\mathcal{E}$, reward function r, and an RL agent. At every step a state $s \in \mathcal{S}$ is visited. The virtual agent chooses an action a from all the possible actions included in the action space $\mathcal{A}$. Consequently, the process transitions to state s' and the agent receives a reward $r_a(s, s')$ generated by the environment $\mathcal{E}$. The probability to visit the state s' depends on the chosen action. If s and a are not dependent on earlier states and actions, i.e., the process is memoryless, the Markov property is satisfied, and the reinforcement learning task can be solved as an MDP. A finite MDP is defined as finite sets of states s and actions a, and the transition probability from the state-action pair (s, a) to every possible next state s' [43]. This transition is associated with a reward r and can be expressed by

$$p(s', r|s, a) = \Pr(s_{t+1} = s', r_{t+1} = r|s_t = s, a_t = a), \tag{1}$$

where Pr represents the probability. Consequently, the expected reward $r(s, a)$ of a state-action pair is

$$r(s, a) = E[r_{t+1}|s_t = s, a_t = a] = \sum_r r \sum_{s'} p(s', r|s, a), \tag{2}$$

with the transition probabilities

$$p(s'|s, a) = \Pr(s_{t+1} = s', |s_t = s, a_t = a) = \sum_r p(s', r|s, a). \tag{3}$$

$E[.]$ symbolizes the expected reward. A policy π maps the probability $\pi(a|s)$ to choose a possible action a in the state s. The expected reward achieved by following the policy π in state s. $V_\pi(s)$ is referred to as the value of state s under policy π and can be described by the value function, formally expressed for MDPs as

$$V_\pi(s) = E_\pi[G_t|s_t = s] = E_\pi[\sum_{k=0}^{\infty} \gamma^k r_{t+k+1}|s_t = s]. \tag{4}$$

Analog the value of an action a in state s under policy π can be defined as $Q_\pi(s, a)$. This function is equivalent to the expected reward returned after choosing the action a in state s and the subsequent following of the policy π. This function is called 'action-value function of policy π' and plays a central role for reinforcement learning methods. Using the experience of the agent, $V_\pi(s)$ and $Q_\pi(s, a)$ can be estimated as

$$Q_\pi(s, a) = E_\pi[G_t|s_t = s, a_t = a] = E_\pi[\sum_{k=0}^{\infty} \gamma^k r_{t+k+1}|s_t = s, a_t = a]. \tag{5}$$

A fundamental property of these value functions, which is used for reinforcement learning and dynamic programming, is the recursive relation between $V_\pi(s)$ and $V_\pi(s')$, as expressed by the Bellman equation:

$$
\begin{aligned}
V_\pi(s) &= E_\pi[G_t|s_t = s] \\
&= E_\pi[\sum_{k=0}^{\infty}\gamma^k r_{t+k+1}|s_t = s] \\
&= E_\pi[r_{t+1} + \gamma\sum_{k=0}^{\infty}\gamma^k r_{t+k+2}|s_t = s] \\
&= \sum_a \pi(a|s)\sum_{s'}\sum_r p(s', r|s, a)[r + \gamma E_\pi[\sum_{k=0}^{\infty}\gamma^k r_{t+k+1}|s_t = s']] \\
&= \sum_a \pi(a|s)\sum_{s',r} p(s', r|s, a)[r + \gamma V_\pi(s')],
\end{aligned} \tag{6}
$$

which shows the relation between the value function of state s and it's possible following states s'. The Bellman equation is a sum of three variables a, s' and r. For every triple, the probability $p(s', r|s, a)$ is calculated and summed up, where $\pi(a|s)$ represents the policy [43].

1.1.1 Q-Learning: Off-Policy TD

Based on the Bellman equation, the simplest form of Q-learning can be derived:

$$Q(s_t, a_t) \leftarrow Q(s_t, a_t) + \alpha[r_{t+1} + \gamma \max_a Q(s_{t+1}, a) - Q(s_t, a_t)]. \tag{7}$$

In this case, the learned action-value function Q is directly approximated from Q_*, the optimal action-value function, and is therefore independent of the policy. Q-learning is off-policy learning because the action selection policy differs from target policy. Instead of following the target policy, the action with the highest Q-value is always selected, which corresponds to a maximum ϵ-greedy policy. The target policy still affects it by selecting which state-action pairs are visited and updated.

1.2 Actor-critic methods

Actor-critic methods are TD methods with a separate memory structure for representing the policy. This policy structure is called 'actor' because it selects the actions. In contrast, the estimated value function is the 'critic', because this evaluates the actions that the actor chooses. The critic, on the other hand, learns and judges the currently executing policy, which is in the form of a TD error. This scalar signal, which is calculated after every action, is the only output of the critic and causes the learning process for both the actor and the critic and can be formally described by

$$\delta_t = r_{t+1} + \gamma V_t(s_{t+1}) - V(s_t), \tag{8}$$

where V_t is the value function implemented by the critic at time t. For many variations of the Actor-critic algorithm, the action value function $Q_t(s_t, a_t)$ is used instead of the state value function $V_t(s_t)$ to calculate δ_t. If the TD error is positive, the tendency to choose action a_t should be strengthened, whereas if the TD error is negative, it should be weakened.

One step to further improve the algorithm is to replace the discounted reward with an average reward; this off-policy method is called 'R-learning'. This method aims at a maximum average reward per time step. The value function for a policy π is defined similarly to the average expected reward per time step $\overline{r}(\pi)$:

$$\overline{r}(\pi) = \lim_{n\to\infty} \frac{1}{n} \sum_{t=1}^{n} E_{\pi}[R_t]. \tag{9}$$

Equation 9 satisfies the recurrence of a Markov chain, see equation 1. This means that after several episodes the average reward starting from any state remains the same and thus the value function can be formulated as follows:

$$V_{\pi}(s) = \sum_{k=1}^{\infty} E_{\pi}[R_{t+k} - \overline{r}(\pi) \,|\, s_t = s]. \tag{10}$$

The state-action function can be written in the same way:

$$Q_{\pi}(s,a) = \sum_{k=1}^{\infty} E_{\pi}[R_{t+k} - \overline{r}(\pi) \,|\, s_t = s, a_t = a]. \tag{11}$$

Actor-critic methods implement the average reward, or 'advantages', are often referred to in the literature as "'Advantage Actor-critic"' (AAC or A2C).

2 Methods

2.1 Casting the AmsterdamUMCdb as an RL environment

The basic structure of the environment was adapted to that of an OpenAI Gym Environment: [44].

The input of the environment corresponds to the action $a_t \in \mathcal{A}$ and is an integer in $[0, 4]$ for this application. The environment's output consists of the current state s_t of the environment at time t, the reward r_t at time t and a boolean variable $done_t$, which indicates whether the environment has reached the final state and an additional informational variable. For this application, the state s_t corresponds to the 379 medical parameters and comprises the available health data of patient at one day. The reward r_t represents the physiological feedback of the patient to the treatment and is also derived from the measured data.

The database was transformed into a functional environment as follows: For a retrospective analysis of the impact of cortisone administration to septic patients, only measured state trajectories can be considered. Therefore, the input action $a_{RL,t}$ is ignored in this environment. Instead the action $a_{C,t}$ corresponding to the human clinicians' treatment during the data collection is chosen, hence the environment must only visit known states. This implies that the action $a_{C,t}$ actually chosen to treat each patient is always selected to visit only known (measured) states. For this reason, an off-policy algorithm is necessary since the agent's updated policy does not determine its behavior: Often, this problem is circumvented by a clustering algorithm by grouping similar patient states into approximately 100 to 1000, depending on the size of the data set. Then, transition probabilities can be calculated by

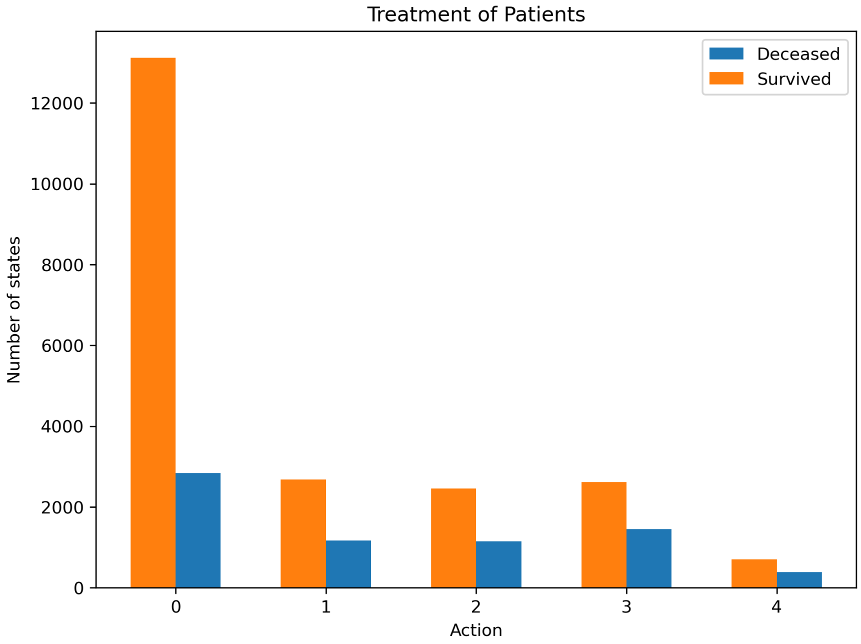

Figure 1: Distribution of the states depending on the clinician‘s action and mortality

rasterizing the entire data set and counting how often a state has transitioned to another state depending on the selected action. Thus, an environment can be created from a data set that can find a state for any action. This method was deliberately not chosen for this work. By combining off-policy updates with a stable stochastic actor-critic formulation, the RL framework can also learn from the clinicians' actions [45]. Due to this restriction, the visitable states are significantly reduced, as shown in figure 1. However, the algorithm can learn with the actual measured values and the input state does not require a reduction in its dimension. Therefore, the agent's decision can be interpreted more easily, since, for in-stance, the relevance of the individual input features can be directly analyzed. Furthermore, it should also be noted that clustering the states does not solve the problem of the limited number of states. By reducing the problem's dimensionality, other inaccuracies arise instead of the problem of missing states: The assignment of a real state to the reduced state is not exact. Moreover, the correctness of the transition probabilities also depend on the number of measured states. Figure 2 and 3 show the result of two different clustering algorithms with the used data set: From these figures, it is evident that the clinician's decision correlates weakly with clustering. This uncertainty would significantly impact the final quality of the reinforcement learning algorithm.

The choice of the reward signal also plays an important role in creating a reliable RL algorithm. Several reward functions were tested, all based on a combination of mortality and SOFA score. The following functions were tested:

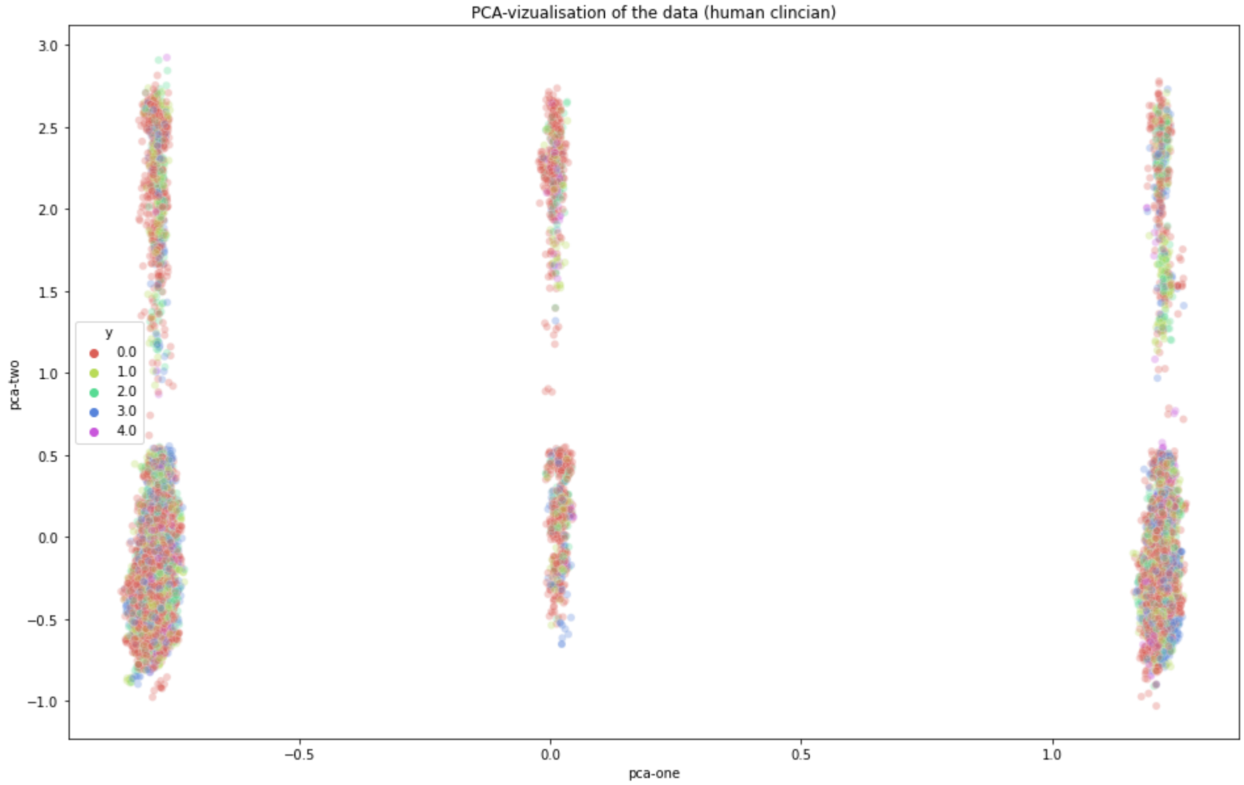

Figure 2: This figure shows the result of the Principal Component Analysis (PCA) clustering algorithm using the data set; the states have been colored depending on the clinician's action.

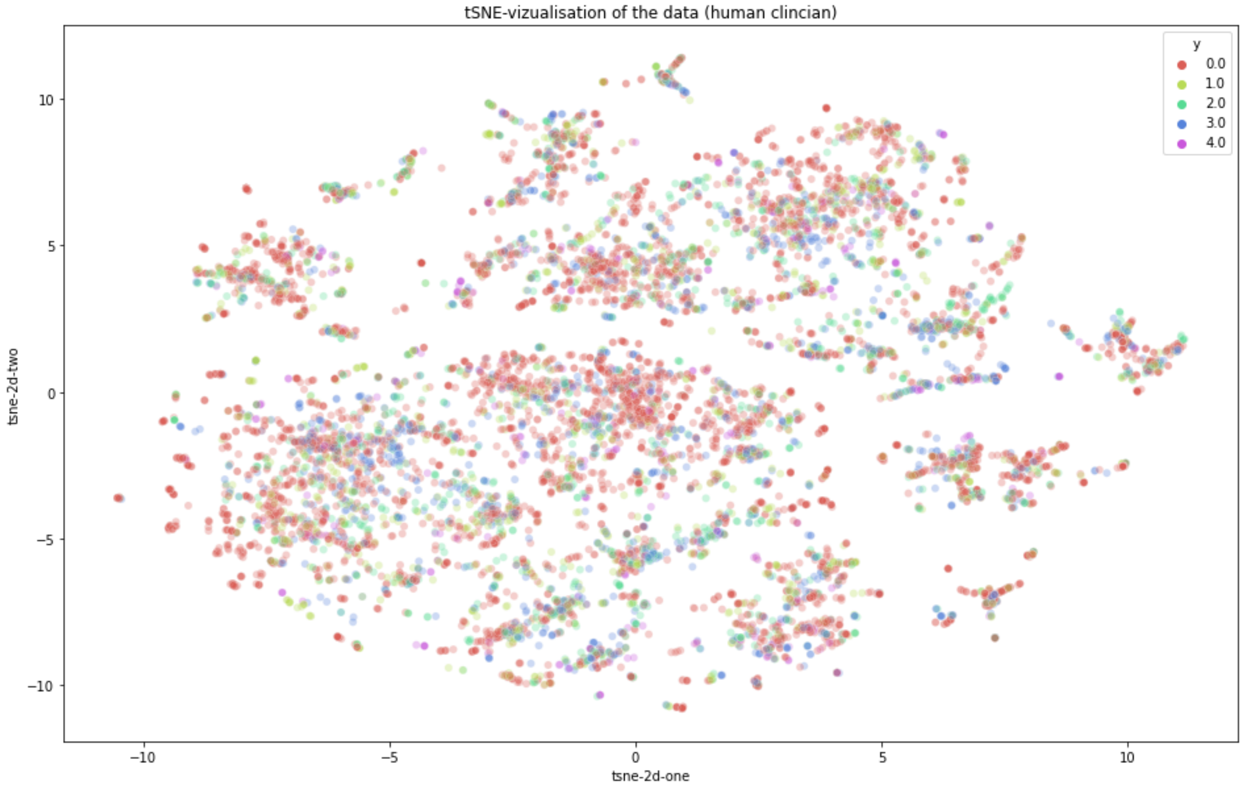

Figure 3: This figure shows the result of the t-distributed stochastic neighbor embedding-clustering (tSNE) algorithm using the data set; the states have been colored depending on the clinician's action.

$$
\begin{aligned}
&r_t = 1 - b_{\text{mort}},\\
&r_t = 1 - \text{SOFA}_t/\text{SOFA}_{\max},\\
&r_t = \max(1 - \text{SOFA}_t/\text{SOFA}_{\max} - b_{\text{mort}}, 0),\\
&r_t = 1 - \text{SOFA}_{t+1}/\text{SOFA}_{\max},\\
&r_t = \max(1 - \text{SOFA}_t/\text{SOFA}_{\max} - b_{\text{mort}}, 0),\\
&r_t = -(\text{SOFA}_{t+1} - \text{SOFA}_t)/\text{SOFA}_{\max},\\
&r_t = \max(-(\text{SOFA}_{t+1} - \text{SOFA}_t)/\text{SOFA}_{\max} - b_{\text{mort}}, 0),\\
&\text{if: } ((\text{SOFA}_{t+1} - \text{SOFA}_t) < 0) : r_t = 1; \quad \text{else: } r_t = 0,
\end{aligned}
\tag{12}
$$

where $b_{\text{mort}} = 1$ if the patient deceased and $b_{\text{mort}} = 0$ if the patient survived. We have tested $b_{\text{mort}} = 1$ as a constant and time-dependent variable depending on the patient and concluded to leave b_{mort} only depending on the patient ID, as we concluded that it is better to lower the reward of the whole treatment episode. In addition, we also tested these reward signals without the max functions. The basic idea of the environment is that an internal pointer rasterizes the data points, each representing a patient state on a day: for this purpose, the patients were assigned IDs and then these IDs were randomly assigned to the test set, the validation set, and the training set. At the beginning of the training, the environment is reset and thus, the pointer is set to the first day of the patient in the ICU in the training set and its state is returned. When the environment transitions to the next state, the pointer simply moves to the next line, then it checks if the patient ID changes in the next step: If it does, *done* is set to $True$ and the pointer is set to the initial value of another random patient that has not yet occurred in the training set. If not, *done* is set to $False$ and the next patient state s_t is returned. The agent can retrieve the clinician's action at that pointer location by another class function.

2.2 Network architecture and hyperparameter selection

In the first phase of the project, we tested with other stable baseline algorithms, but due to the increased freedom, we used a custom soft-actor critic algorithm, where a neural network represents the policy of the reinforcement algorithm. Since the individual input parameters consist of independent numerical values, a relatively simple network architecture can be chosen. Therefore, a network consisting of 256 neurons in the hidden layer was used; 256 was chosen because it is between the number of input and output features. For both the actor and the critic model, the same network architecture was used; they differ only in the output layer: the actor has five outputs, representing the action space, the critic only one. Initially, there was an intensive discussion regarding the optimal action space. Initial tests had more than five possible actions, but we reduced the number of actions since the clinical experts on our team felt that the differences between the actions would be too small at smaller intervals. Even with the final chosen distribution of actions, the agreement among human physicians would already be low at actions > 0. For the optimizer, RMSprop was chosen since it is shown to be effective with gradient descent methods. The actor and critic model were trained simultaneously on the training set: The critic loss function is based on the mean squared error loss; the actor model was optimized to a loss function, which is based

on equation 7, categorical cross-entropy loss. We optimized the models on various numbers of neurons, batch sizes, and learning rates and also did some tests with different gamma values. These parameters have been chosen for the final model:

Number of neurons	256
Batch size	512
Learning rate	0.00008
Gamma	0.99

The validation set was used mainly for calculating the HCOPE lower bound value. To avoid overfitting leading to high HCOPE values, we performed HCOPE only on unknown data. About 1000 models were trained and tested for each reward signal. The best model was selected based on the highest HCOPE value achieved.

2.3 Training

Training Algorithm 1 shows the actor-critic model in more detail: After initializing step counter t and episode counter E, the algorithm repeats until the $E > E_{max}$. At the start of every episode, the gradients get reset to 0 and t_{start} is set to the current time step t. Subsequently, the agent receives the first state s_t from the environment, which is the feature vector extracted from the data set. Then following steps are looped until $t == t_{final}$, where t_{final} is the batch size: The actor acts according to the clinician's policy $\pi_C(a_t|s_t)$. As a result, the environment reacts with a new state s_{t+1} and associated reward r_t . Then the vector $dones[t]$ is set to 0, if st is terminal, else it is set to 1. This provides information about the connection of trajectories, which is essential for calculating the return R. Then the next step starts. After the loop, the critic estimates the next value, so that the return can be set to $R[t+1] = V(s_t; \Theta_v)$. Then a reverse loop from t to t_{start} is used to set the return R and calculate the gradients $d\delta$ and $d\delta_v$. The term $1 - dones[t]$ would reduce the regression to $R[t] \leftarrow r[t]$, if the state s_t were terminal. $R - V(s_t; \Theta_v))$ is called 'advantage' and gives the advantage actor-critic method its name. The update of the actor's weights depends on the chosen actions, which are influenced by the clinician's policy $\pi_C(a_t|s_t)$. The critic's update is based on equation 8. Subsequently, the HCOPE value is calculated, and the model parameters are saved.

The loss, the HCOPE values, and the average reward were observed and saved during the training process. After the loss and the HCOPE value converged, the training was stopped. Subsequently, the model version, which scored the highest HCOPE value during the training process, was selected as the final model for a certain hyperparameter configuration. We concluded that this method reduces overfitting, similar to early-stopping, and obtains the global maximum of the HCOPE value.

2.4 Policy Evaluation

Off-policy evaluation is significantly more complex than on-policy evaluation, because the reward received after each episode reflects the performance of the behavior policy but not the evaluation policy. Therefore, we applied various methods to ensure that the policy of the algorithm is still reliably evaluated: The first method was applied mainly during training

Algorithm 1: Actor-critic - pseudocode

Initialize step counter $t \leftarrow 1$
Initialize episode counter $E \leftarrow 1$
repeat
  Reset gradients: $d\Theta \leftarrow 0$ and $d\Theta_v \leftarrow 0$
  $t_{start} = t$
  Get state s_t from environment
  repeat
    Perform a_t according to clinician's decision $\pi_C(a_t|s_t)$
    Recieve reward r_t and new state s_{t+1}
    if s_t *is terminal* **then**
      $dones[t] = 1$
    end
    else
      $dones[t] = 0$
    end
    $t \leftarrow t + 1$
  until $t == t_{final}$;
  $R[t+1] = V(s_t; \Theta_v)$
  for $i \in \{t, \ldots, t_{start}\}$ **do**
    $R[t] \leftarrow r[t] + \gamma R[t+1] \cdot (1 - dones[t])$
    Update actor's weights: $d\theta \leftarrow d\theta + \alpha_\theta(R - V(s_t; \Theta_v))\nabla_\theta \ln \pi_C(a_t|s_t) + \beta \cdot \partial H(\pi_C(a_t|s_t))/\partial\theta$
    Update critic's weights: $d\theta_v \leftarrow d\theta_v + \beta_v(R - V(s_t; \Theta_v))\partial V(s_t; \Theta_v)/\partial\theta$
  end
  $E \leftarrow E + 1$
  Update θ and θ_v using $d\theta$ and $d\theta_v$
  Calculate HCOPE value and save model parameters
until $E > E_{max}$;

due to its simplicity, in order to better track the learning curve. The principle idea of this evaluation method is to compare the average reward of the actions where AI agent and human clinicians agree with those without agreement. This method can represent the learning process well, but also requires cautious interpretation:

Action 0 is selected more likely for the healthier patients in this dataset. A policy that always chooses action 0 will thus be represented better than the human physician's policy by this evaluation method, if the reward is calculated from mortality only. This is because only matches at action 0 would be found, these patients would have lower average mortality, and thus the matched actions would have a higher reward than the unmatched actions.

The HCOPE value was mainly employed during the training process as a policy evaluation. After importance sampling of the data points, this algorithm can calculate a lower bound for the expected reward by statistically evaluating the received reward depending on the agreement probability. The lower bound was calculated only on the evaluation set to minimize overfitting.

In addition, Layerwise-Relevance Propagation was used to evaluate the relevance of each medical input feature on the final decision of the algorithm. Afterward, a Support Vector Machine was fitted to the actions of the human clinicians to generate a comparable relevance vector representing the human clinicians' behavior. Both relevance vectors were compared and subsequently tested for reasonability by the medical experts in our team. Here, we confirmed that the algorithm mainly focuses on medical features already established in the medical field. However, it also demonstrates that the AI agent considers significantly more parameters than the human doctor, but this was to be expected.

Another method was to analyze specific cases in the existing data set. For example, clinicians administer more cortisone in septic shock than usual. We evaluated whether the algorithm exhibited similar behavior. Additionally, the hospitalization histories of individual random patients were graphed and examined for their medical reasonableness.

Finally, the actions of the AI agent were also observed dependent on features such as weight and gender.

References

[43] Richard S. Sutton and Andrew G. Barto, Reinforcement Learning: An Intro-duction, 2015

[44] Brockman, G. et al., 2016. Openai gym. arXiv preprint arXiv:1606.01540.

[45] Haarnoja, Tuomas and Zhou, Aurick and Abbeel, Pieter and Levine, Sergey, Soft Actor-Critic: Off-Policy Maximum Entropy Deep Reinforcement Learning with a Stochastic Actor, https://doi.org/10.48550/arxiv.1801.01290
