# Supplementary material for: Development of a Reinforcement Learning Algorithm to Optimize Corticosteroid Therapy in Critically Ill Patients with Sepsis
Source: J Clin Med. 2023 Feb 14;12(4):1513. doi: 10.3390/jcm12041513 (PMC9961939; doi:10.3390/jcm12041513)
Supplement: Supplementary file 1 [file jcm-12-01513-s001.zip › Supplemental Material.pdf]

Development of a Reinforcement Learning Algorithm to Optimize Corticosteroid Therapy in Critically Ill Patients with Sepsis

Supplemental Material. Table of Contents

Supplemental Material. Table of Contents 1

Supplemental Table S1. Diagnosis of sepsis 2

Supplemental Table S2. Input features included in development of the algorithm 3

Supplemental Figure S1. Development of the RL Algorithm 5

Supplemental Figure S2. Micro-average ROC curve of the random forest model 6

Supplemental Table S3. The most relevant predictors of the clinicians' policy according to the random forest model ordered from the lowest to highest rank 7

Supplemental Table S4. The most relevant feature for the RL policy listed from the lowest to highest rank 8

Supplemental Figure S3. The 20 most relevant input features for the RL and random forest models 9

Supplemental Table S1. Diagnosis of sepsis

Non-prophylactic anti-infective drugs used	Cultures drawn suggestive of sepsis	Admission diagnosis suggestive of infection
Amikacin	Urine culture	Pneumonia
Amoxicillin	MRSA swab	Meningitis
Benzylpenicillin	Blood culture	Endocarditis
Ceftazidime	Catheter tip culture	Cholangitis
Cefotaxime	Drain fluid culture	Pancreatitis
Ciprofloxacin	Stool culture	Abscess
Rifampicin	CSF culture	Fasciitis
Clindamycin	Nasal swab	Peritonitis
Tobramycin	Perineal swab	GI perforation/rupture
Vancomycin	Rectal swab	GI ischemia
Imipenem	Wound swab	Diverticulitis
Doxycycline	Ascites culture	Sepsis
Metronidazole	Legionella urinary antigen	Infection
Erythromycin		Inflammatory
Flucloxacillin		
Fluconazole		
Ganciclovir		
Flucytosine		
Gentamicin		
Foscarnet		
Amphotericin B		
Meropenem		
Myambutol		
Co-Trimoxazole		
Voriconazole		
Amoxicillin/Clavulanic acid		
Aztreonam		
Chloramphenicol		
Fusidic acid		
Piperacillin		
Ceftriaxone		
Cefuroxime		
Cefazoline		
Caspofungin		
Itraconazole		
Levofloxacin		
Anidulafungin		
Linezolid		
Tigecycline		
Daptomycin		
Colistin		

MRSA: Methicillin-resistant Staphylococcus aureus; CSF: cerebrospinal fluid; GI: gastro-intestinal.

Patients with sepsis were identified based on the Sepsis-3 criteria. Accordingly, patients with new organ dysfunction as indicated by either a SOFA score ≥2 at admission or an increase of 2 points or more in the SOFA score during the ICU stay in the context of suspected infection were included in the sepsis cohort used to develop the RL algorithm. The definition of suspected infection, which has been previously described (Thoral et al., AmsterdamUMCdb GitHub repository), was operationalized by identifying antibiotic therapy (other than prophylactic use), cultures drawn, sepsis flagged by admitting physicians or admission diagnosis suggestive for severe infection. The onset of the septic episode was considered the day the change in the SOFA score occurred and patients remained in the sepsis cohort until discharge or death.

Supplemental Table S2. Input features included in development of the algorithm

Category	Variable	Type	Preprocessing and derived features
**Patient characteristics**	Age (years)	Discrete	Bins*
	Male gender	Boolean	-
	Weight (kg)	Continuous	-
	Admission count	Discrete	-
**Vital parameters**	Respiratory rate (min^{-1})	Continuous	Mean, minimum, maximum, standard deviation
	Heart rate (min^{-1})	Continuous	
	Invasive systolic, diastolic, and mean blood pressure (mmHg)	Continuous	
	Non-invasive systolic, diastolic, and mean blood pressure (mmHg)	Continuous	
	SpO_2	Continuous	
	Temperature (°c)	Continuous	
**Laboratory values**	AG (mEq/l), BE (mEq/l), Bicarbonate (mEq/l), pH, Lactate (mmol/l), $PaCO_2$ (mmHg), PaO_2 (mmHg), SaO_2	Continuous	Mean, minimum, maximum, standard deviation
	ACTH (pmol/l), Cortisol (nmol/l), TSH (mIU/l), fT_3 (pmol/l)	Continuous	
	Albumin (g/l), Ammonia (µmol/l), Bilirubin (µmol), GOT (U/l), GPT (U/l)	Continuous	
	Blood glucose (mmol/l)	Continuous	
	CRP (mg/l), PCT (ug/l)	Continuous	
	Ca (mg/dl), Cl (mmol/l), Fe (µmol/l), K (mmol/l), Mg (mg/dl), Na (mmol/l), Phosphate (mg/dl), *i*Ca	Continuous	
	Total cholesterol, HDL-cholesterol, LDL-cholesterol (mmol/l) Triglycerides (mg/dl)	Continuous	
	Serum creatinine (µmol/l), Serum urea (mmol/l), eGFR (ml/min)	Continuous	
	Fibrinogen (mg/dl), PTT (s), PT (s)	Continuous	
	GSF Glucose (mmol/l), CSF Leucocytes (ml^{-1}), CSF Protein (mg/dl)	Continuous	

	Hematocrit, Hemoglobin (g/l), RBC count, Leucocyte count, Lymphocytes count, Neutrophils count, MCH (pg), MCV (fl), Thrombocyte count	Continuous	
	Urinary Na (mEq/l), Urinary K (mEq/l), Urinary Creatinine (mmol/day), Urinary Urea (mmol/day)	Continuous	
**Ventilation parameters**	FiO_2	Continuous	Mean, minimum, maximum, standard deviation
	PEEP (cmH_2O)	Continuous	
	Set respiratory rate (min^{-1})	Continuous	
**Fluid balance**	Urine output (ml)	Continuous	-
	Net fluid balance (ml)	Continuous	-
	Ultrafiltration rate (ml/h)	Continuous	-
**Commonly used medication**	Dose of fast-acting insulins: Actrapid, Novorapid, Velosulin (IU)	Continuous	Mean, minimum, maximum, standard deviation, and sum for continuously administered drugs
	Dose of benzodiazepines: Alprazolam, Lorazepam, Midazolam, Oxazepam, Temazepam	Continuous	
	Dose of other sedatives and analgesics: Clonidine, Fentanyl, Haloperidol, Morphine, Propofol	Continuous	
	Dose of antiplatelet and anticoagulant drugs: Clopidogrel, Heparin	Continuous	
	Dose of antiarrhythmic drugs: Metoprolol, Amiodarone	Continuous	
	Dose of vasopressors and inotropic agents: Digoxin, Dopamine, Noradrenaline	Continuous	
	Diuretics: Furosemide, Spironolactone	Continuous	
	Highest antibiotic rank**	Discrete	
	Antiviral drugs	Boolean	
	Antifungal drugs	Boolean	

Supplemental Table S2 presents all the input variables collected and the derived features used for developing the reinforcement learning algorithm, after excluding the variables not represented < 2% of the datapoints. SpO2: peripheral oxygen saturation; SOFA: sequential organ failure assessment; AG: anion gap; BE: base excess; pH: potential of hydrogen; $PaCO_2$, PaO_2: partial pressure of carbon dioxide and oxygen, respectively, in arterial blood; SaO_2: arterial oxygen saturation; ACTH: adrenocorticotropic hormone; TSH: thyroid-stimulating hormone; fT_3: free triiodothyronine; GOT: serum glutamic-oxaloacetic transaminase; GPT: serum glutamic-pyruvic transaminase; CRP: C reactive protein; PCT: procalcitonin; Ca: total calcium; Cl: chloride; Fe: serum iron; K: serum potassium; Mg: serum magnesium; Na: serum sodium; *i*Ca: ionized Calcium; HDL: high-density lipoprotein; LDL: low-density lipoprotein; eGFR: estimated glomerular filtration rate; PTT: partial thromboplastin time; PT: prothrombin time; CSF:

cerebrospinal fluid; RBC: red blood cell; MCH: mean corpuscular hemoglobin; MCV: mean corpuscular volume; FiO_2: fraction of inspired oxygen.

*Age was sorted into bins: 18-39 years, 40-49 years, 50-59 years, 60-69 years, 70-79 years, 80+ years

**Antibiotics were classified by rank after Braykov et al. and the highest rank of antibiotics administered was used as input feature.

Supplemental Figure S1. Development of the RL Algorithm

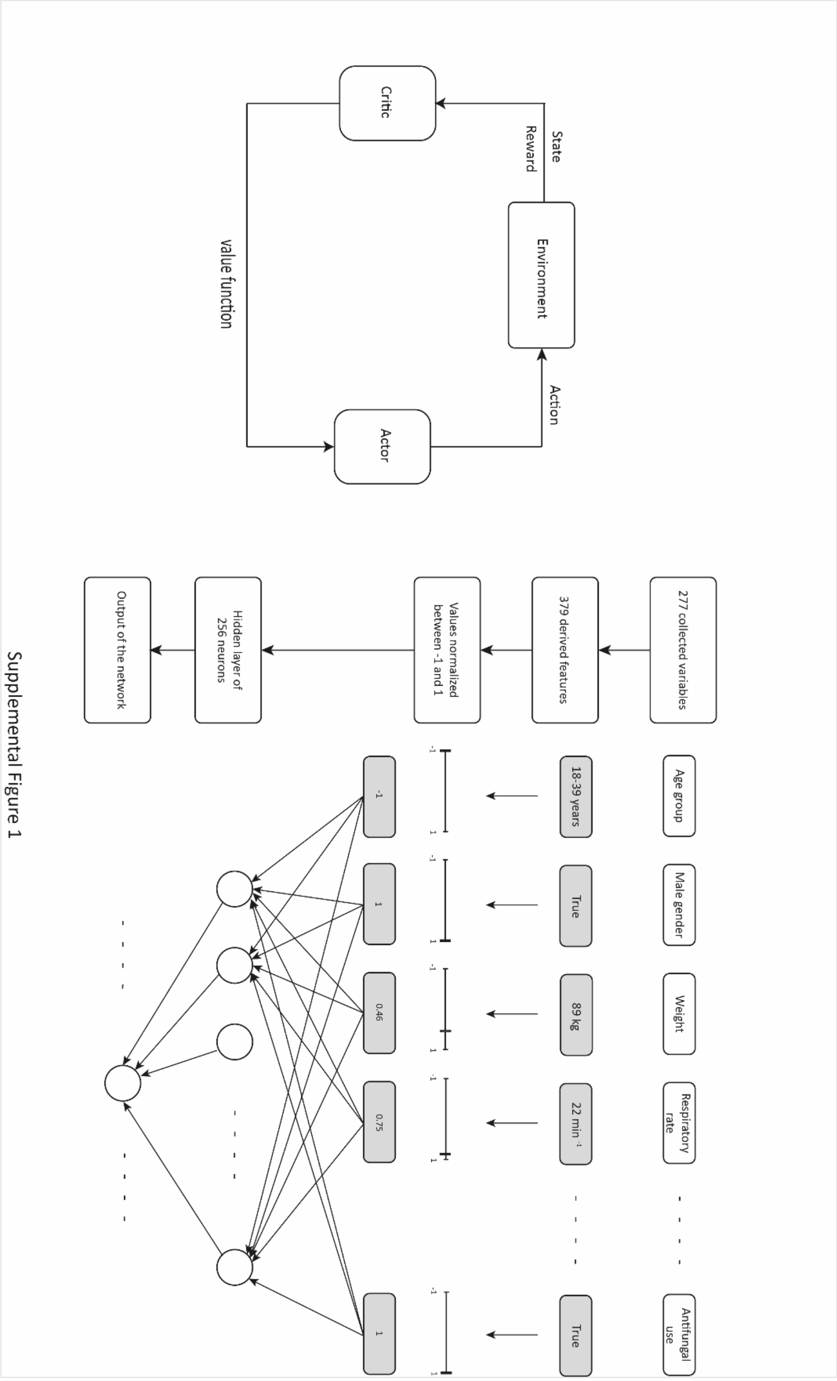

For each day of the ICU stays included in the sepsis cohort, a set of 281 variables were collected, of which 277 were used as inputs. Using imputation and normalization, we derived a balanced dataset of

379 input variables. The RL algorithm consisted of 2 neural networks, with a similar structure that includes a hidden layer of 256 hidden neurons, but different outputs. The actor network has 5 potential outputs, corresponding to the 5 possible actions. The critic network ends in one node terminates with a single node. The 2 networks interact to determine the optimal policy. the actor network proposes an action based on the current state of the environment and the environment changes its state. The critic network evaluates the actor network based on the reward that the chosen action returns.

Supplemental Figure S2. Micro-average ROC curve of the random forest model

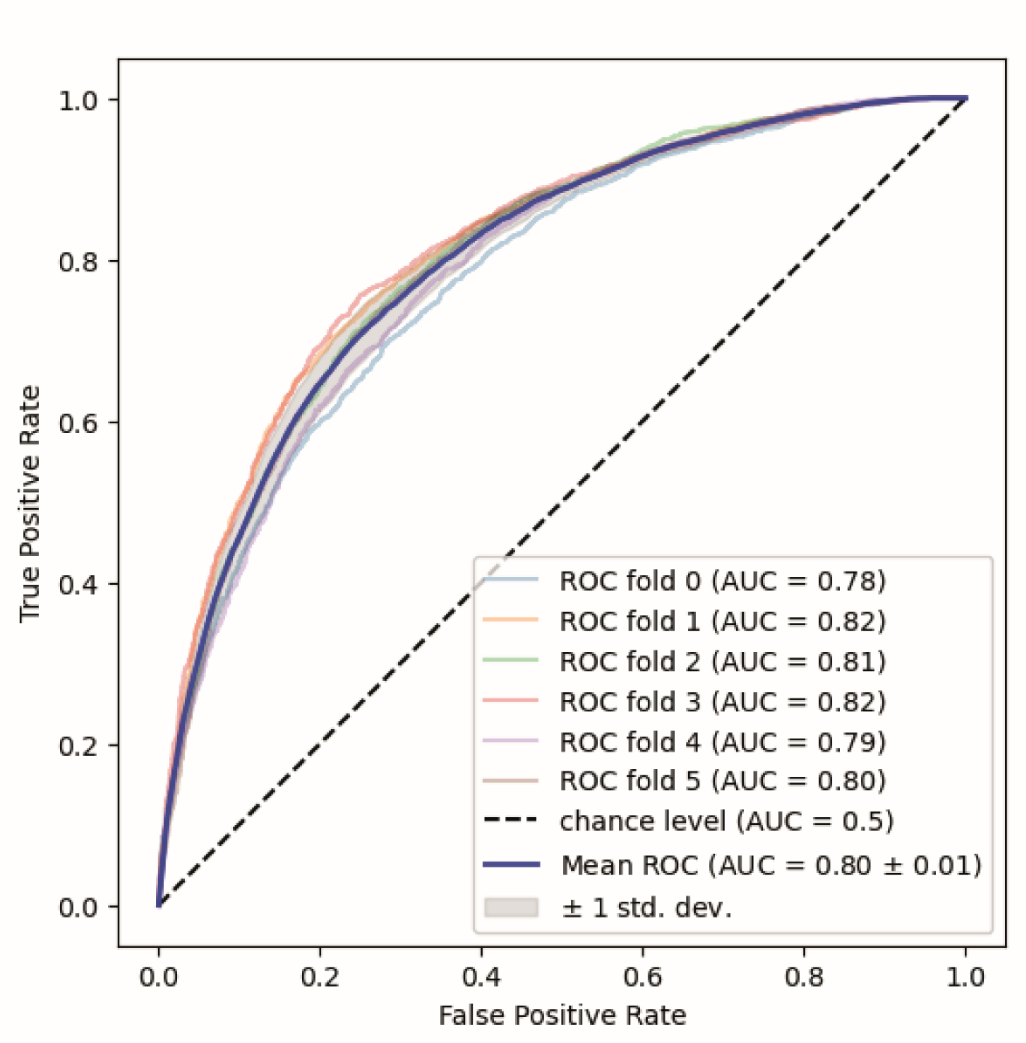

Supplemental Figure 2

The micro-average multiclass AUROC for the random forest model was 0.8. Other performance metrics for the random forest model were:

True positive rate (TPR): 0.7936205665317781

True negative rate (TNR): 0.9255359157578037

Positive predictive value (PPV: 0.8938337801608579

Negative predictive value (NPV): 0.8502332008982553

False positive rate (FPR): 0.07446408424219632

False negative rate (FNR): 0.20637943346822185

False discovery rate (FDR): 0.1061662198391421

Accuracy: 0.8673179955877718

F1-score: 0.8407514815281806

F2-score: 0.8718163275979292

Supplemental Table S3. The most relevant predictors of the clinicians' policy according to the random forest model ordered from the lowest to highest rank

**Feature**	**Component of the unit vector (normalized vectors)**
PTT min	0.124653211
Fentanyl max	0.128485455
Blood glucose max	0.134108057
Length of stay	0.137129421
Thrombocytes min	0.139307112
Urea max	0.142143406
Leucocytes mean	0.142262137
PEEP mean	0.14305812
Blood glucose std	0.147403945
Midazolam (Dormicum) max	0.149040607
PEEP max	0.167538026
PEEP min	0.16780183
PTT max	0.175419476
PTT mean	0.17657839
Highest antibiotic rank	0.177066982
Thrombocytes mean	0.180251789
Noradrenaline (Norepinephrine) sum	0.187155279
Leucocytes max	0.202227827
Thrombocytes max	0.274195479
Noradrenaline (Norepinephrine) max	0.379869603

The normalized vector is the unit vector with a length of 1, which is defined by

$u_e = \frac{u}{||u||}$, where u_e is the normalized vector, u the original vector, and $|u|$ the norm of vector u. The norm of a vector is defined by $||u|| = \sqrt{u_1^2 + u_2^2 + \cdots + u_n^2}$, where u_i is an entry of the vector.

The normalized vector indicates how strongly a single input feature affects the decision in comparison to the other features.

Supplemental Table S4. The most relevant feature for the RL policy listed from the lowest to highest rank

**Feature**	**Component of the unit vector (normalized vectors)**
Velosulin (Insulin) max	0.078966455
Urinary sodium std	0.079285593
Serum sodium max	0.079325068
CSF protein std	0.079650287
CSF protein mean	0.079713876
Magnesium mean	0.081478474
invasive mean BP min	0.081885501
Midazolam max	0.084812754
Leucocytes max	0.086287831
Serum sodium mean	0.088578701
invasive diastolic BP min	0.10005845
Respiratory Rate min	0.105951594
Blood glucose max	0.113368019
Blood glucose std	0.114456484
Heartrate std	0.114888807
Blood glucose mean	0.119304028
Leucocytes mean	0.120227263
Leucocytes min	0.127948494
invasive mean BP mean	0.131318385
invasive diastolic BP mean	0.136660705

PTT: partial thromboplastin time; *i*Ca: ionized Calcium; PaCO2: partial pressure of carbon dioxide in arterial blood; CSF: cerebrospinal fluid; BP: blood pressure; GOT: serum glutamic-oxaloacetic transaminase; GPT: serum glutamic-pyruvic transaminase; PCT: procalcitonin; K: serum potassium; iO2: fraction of inspired oxygen.

The normalized vector is the unit vector with a length of 1, which is defined by

$u_e = \frac{u}{||u||}$, where u_e is the normalized vector, u the original vector, and $|u|$ the norm of vector u. The norm of a vector is defined by $||u|| = \sqrt{u_1^2 + u_2^2 + \cdots + u_n^2}$, where u_i is an entry of the vector.

The normalized vector indicates how strongly a single input feature affects the decision in comparison to the other features.

Supplemental Figure S3. The 20 most relevant input features for the RL and random forest models

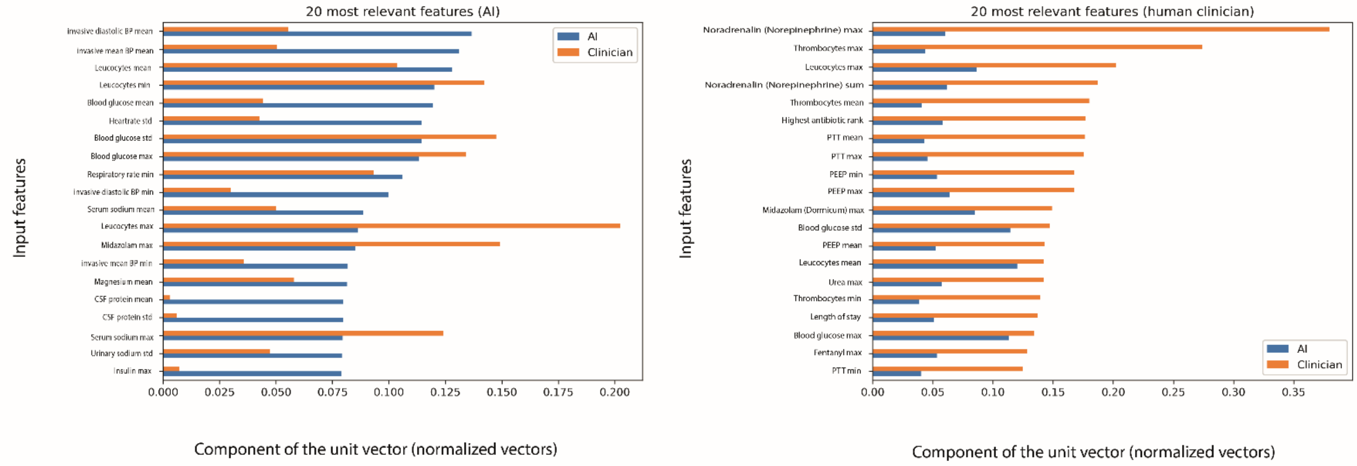

Supplemental Figure 3

The normalized vectors for 20 most relevant features for each model, sorted by rank, are displayed together with the normalized vectors of the same features in the other model.
